# Supplementary material for: Intrachromosomal Looping and Histone K27 Methylation Coordinately Regulates the lncRNA H19-Fetal Mitogen IGF2 Imprinting Cluster in the Decidual Microenvironment of Early Pregnancy
Source: Cells. 2022 Oct 5;11(19):3130. doi: 10.3390/cells11193130 (PMC9563431; doi:10.3390/cells11193130)
Supplement: Supplementary file 1 [file cells-11-03130-s001.zip › cells-1864255-supplementary.pdf]

Supplemental Figures

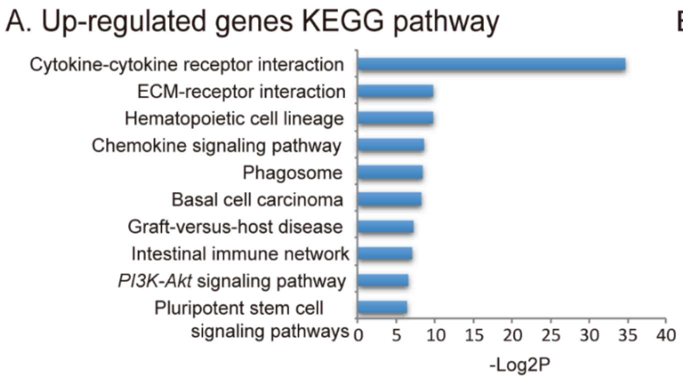

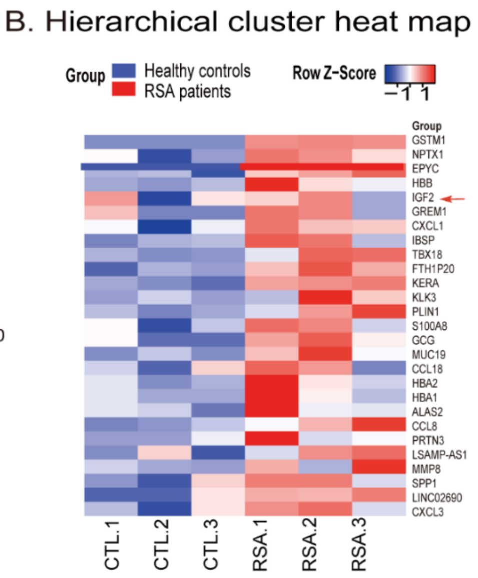

C. Expression of IGF2 (RNA-seq)

IGF2 \ ID	CTL1	CTL2	CTL3	RSA1	RSA2	RSA3
Log2 Counts	9.79	4.28	8.39	8.81	10.10	6.59
Mean ± SD	7.49±2.87			8.50±1.78		

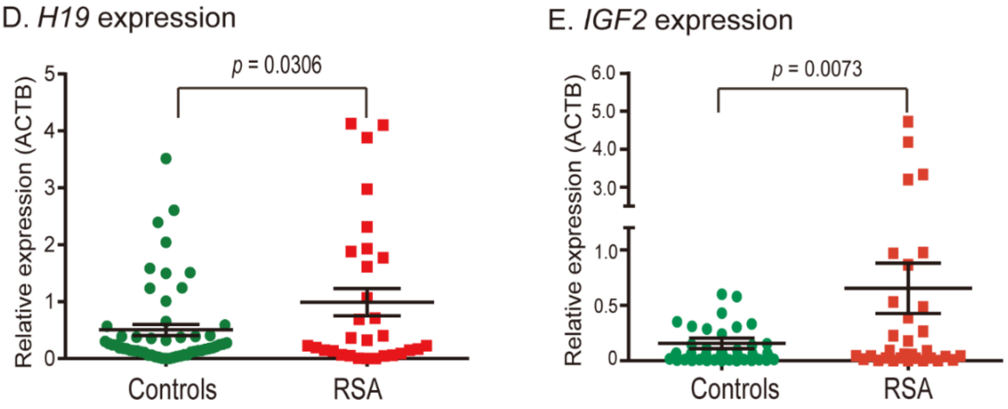

Figure S1. KEGG pathway analysis in RSA patients by RNA-seq. KEGG and hierarchical cluster heat map analyses of RNA-seq data from RSA decidual tissues. **(A)** The Kyoto Encyclopedia of Genes and Genomes (KEGG) pathway analysis of up-regulated protein coding genes (log2FoldChange > 1 or < -1, $p < 0.05$). **(B)** Hierarchical cluster heat map of top differentially expressed RNAs in decidual tissues between RSA patients and controls (log2FoldChange > 2, VALUE<0.001). *IGF2* (arrow) was the sixth candidate gene in the list. **(C)** RNA-seq counts of *IGF2* in the RSA and control samples. The GSE178535 RSA dataset contained the RNA-seq data of decidual tissues from three RSA patients and three healthy control subjects. RNA-seq counts: log2FoldChange. **(D)** *H19* expression in decidual tissues using β-Actin (*ACTB*) as the control. Decidual tissues (32 RSA cases and 57 healthy adult woman controls) were collected. Gene expression was measured by qPCR and standardized over the value of β-Actin (*ACTB*) control. All data shown are mean ± SD. Error bars represent the SE of the average of three independent PCR reactions. $p = 0.03$ as compared with the CTL control. **(E)** *IGF2* expression in decidual tissues using β-Actin (*ACTB*) as the control. Reciprocal upregulation of the *IGF2* mitogen was noticed in decidual tissues of RSA cases. $p = 0.007$ as compared with the CTL control.

A. Imprinting of *H19*

	Imprinting of *H19*		Total	χ^2
	Loss	Maintenance		
Cases	4	17	21	0.407
Controls	6	16	22	
Total	10	33	43	

$P = 0.721$

B. Imprinting of *IGF2*

	Imprinting of *IGF2*		Total	χ^2
	Loss	Maintenance		
Cases	9	14	23	5.258
Controls	4	28	32	
Total	13	42	55	

$P < 0.05$

Figure S2. Aberrant imprinting between the RSA and control groups. The chi-squared analyses of aberrant imprinting between the RSA case and control groups. (**A**) Loss of *H19* imprinting. (**B**) Loss of *IGF2* imprinting.

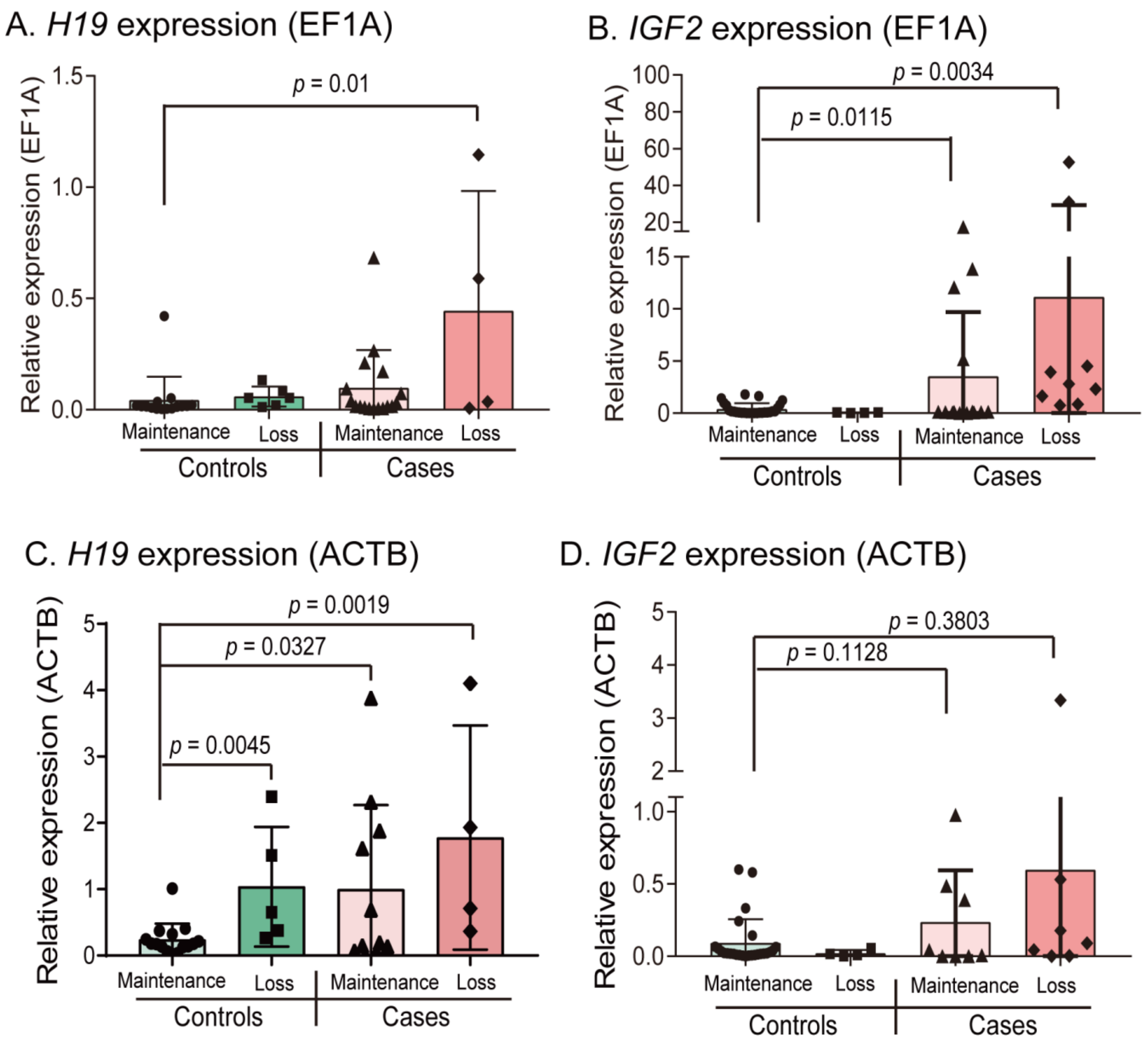

Figure S3. Abundance of *H19*/*IGF2* transcripts between the loss and maintenance subgroups. (**A**) Quantitation of *H19* expression using EF1A (*EEF1A1*) as the qPCR control. (**B**) Quantitation of *IGF2* expression using EF1A (*EEF1A1*) as the qPCR control. (**C**) Quantitation of *H19* expression using β-Actin (*ACTB*) as the qPCR control. (**D**) Quantitation of *IGF2* expression using β-Actin (*ACTB*) as the qPCR control. The data are the mean ± SD from three independent experiments.

A. Culturing of decidual cells

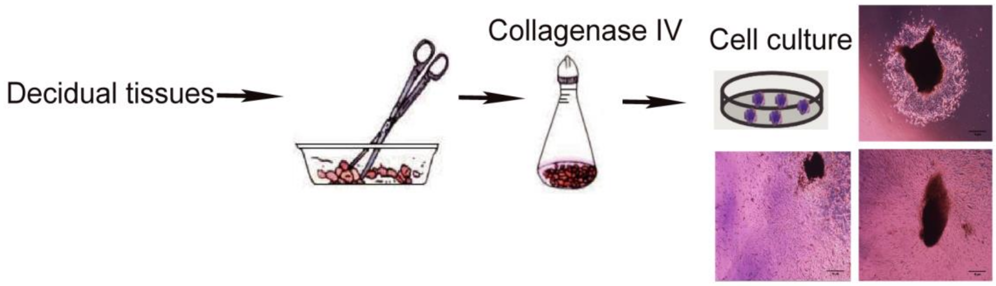

B. Characteristics of two primary endometrial stromal cells

Primary endometrial stromal cells	N45T * (CTL)	U29T ** (RSA)
Patient age (years)	30	32
BMI (cm/kg2)	22	21
Stage	Early pregnancy (7 weeks)	Early pregnancy (8 weeks)
Characteristics	Induced abortion	Recurrent spontaneous abortion
Live births (number)	1	0
Clinical pregnancies (number)	2	4
H19 genotype/cDNA	AT/a (rs3741216)	AC/c (rs2839702)
IGF2 genotype/cDNA	TT/- (rs680)	CT/t (rs680)

Figure S4. Culturing of two primary endometrial stromal cells. (**A**) Schematic diagram of culturing human primary endometrial stromal cells. (**B**) Characteristics of two human primary endometrial stromal cells. * N45T cells are informative for *H19* (AT alleles by genomic DNA typing), and maintain normal imprinting by expressing the "a" allele in its cDNA sample. N45T cells, however, are not informative for *IGF2* (TT alleles) and are thus not suitable for imprinting analysis. ** U29T cells are informative for both *H19* (AC alleles) and *IGF2* (CT alleles). The cDNA analysis shows normal imprinting for *H19* (the c allele) and *IGF2* (the t allele).

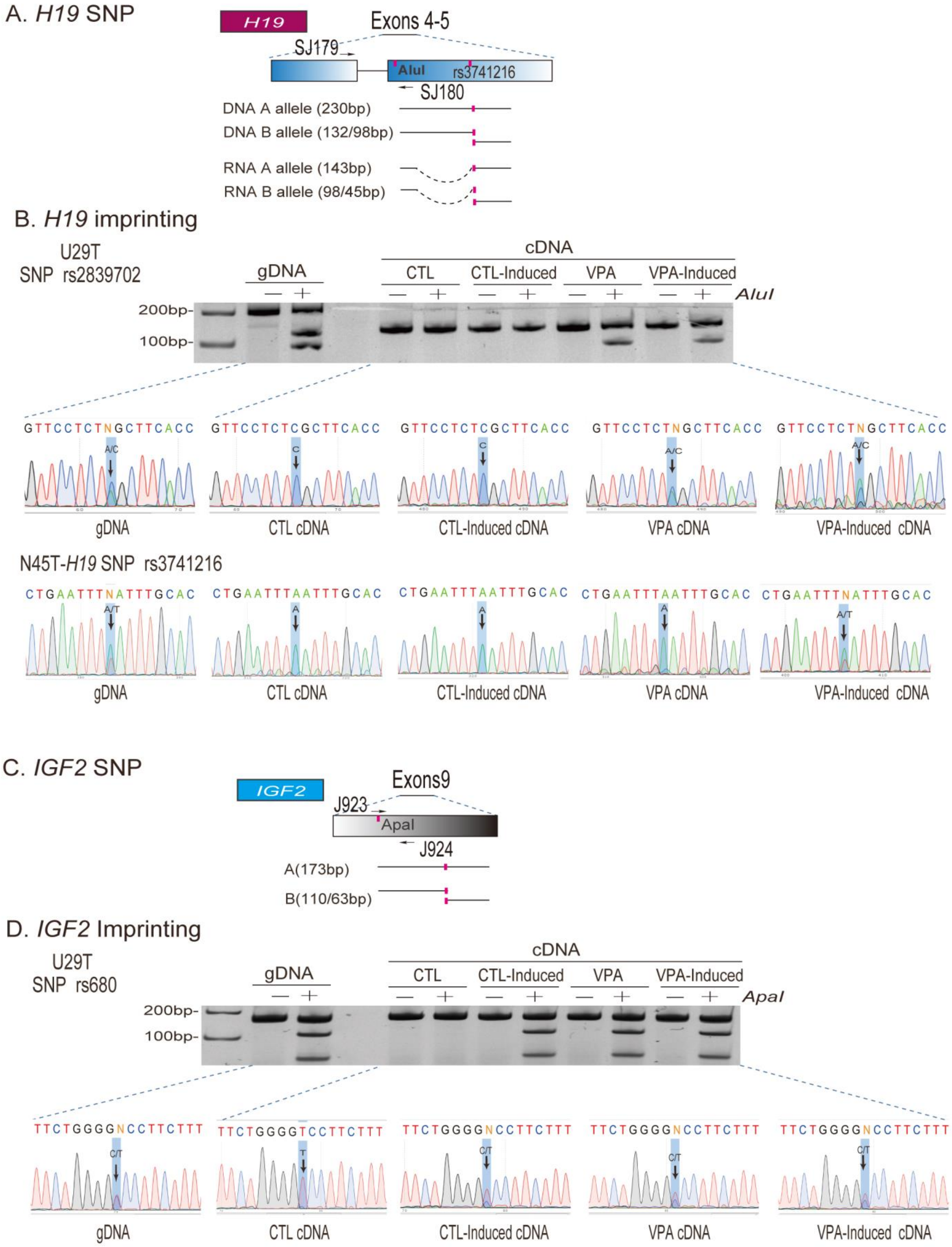

Figure S5. Imprinting after decidualization in VPA-pretreated cells. (**A**) SNP used to map *H19* genotyping and imprinting. (**B**) Loss of *H19* imprinting in VPA-pretreated decidualized U29T and N45T cells. Top panel: Examination of imprinting by Alu1 polymorphic restriction enzyme. Bottom panel: Imprinting by DNA sequencing. Note the loss of *H19* imprinting in VPA-pretreated decidualized U29T and N45T cells (VPA-Induced). Control decidualized U29T and N45T cells (CTL-Induced) still kept normal *H19* imprinting. (**C**) SNP used to map *IGF2* genotyping and imprinting. (**D**) Loss of *IGF2* imprinting in VPA-pretreated decidualized U29T cells. Top panel: Examination of imprinting by Apa1 polymorphic restriction enzyme. Bottom panel: Imprinting by DNA sequencing. Note the loss of *IGF2* imprinting in decidualized U29T cells (both CTL- and VPA-Induced). No informative SNPs are available for N45T cells to distinguish the two parental *IGF2* alleles.

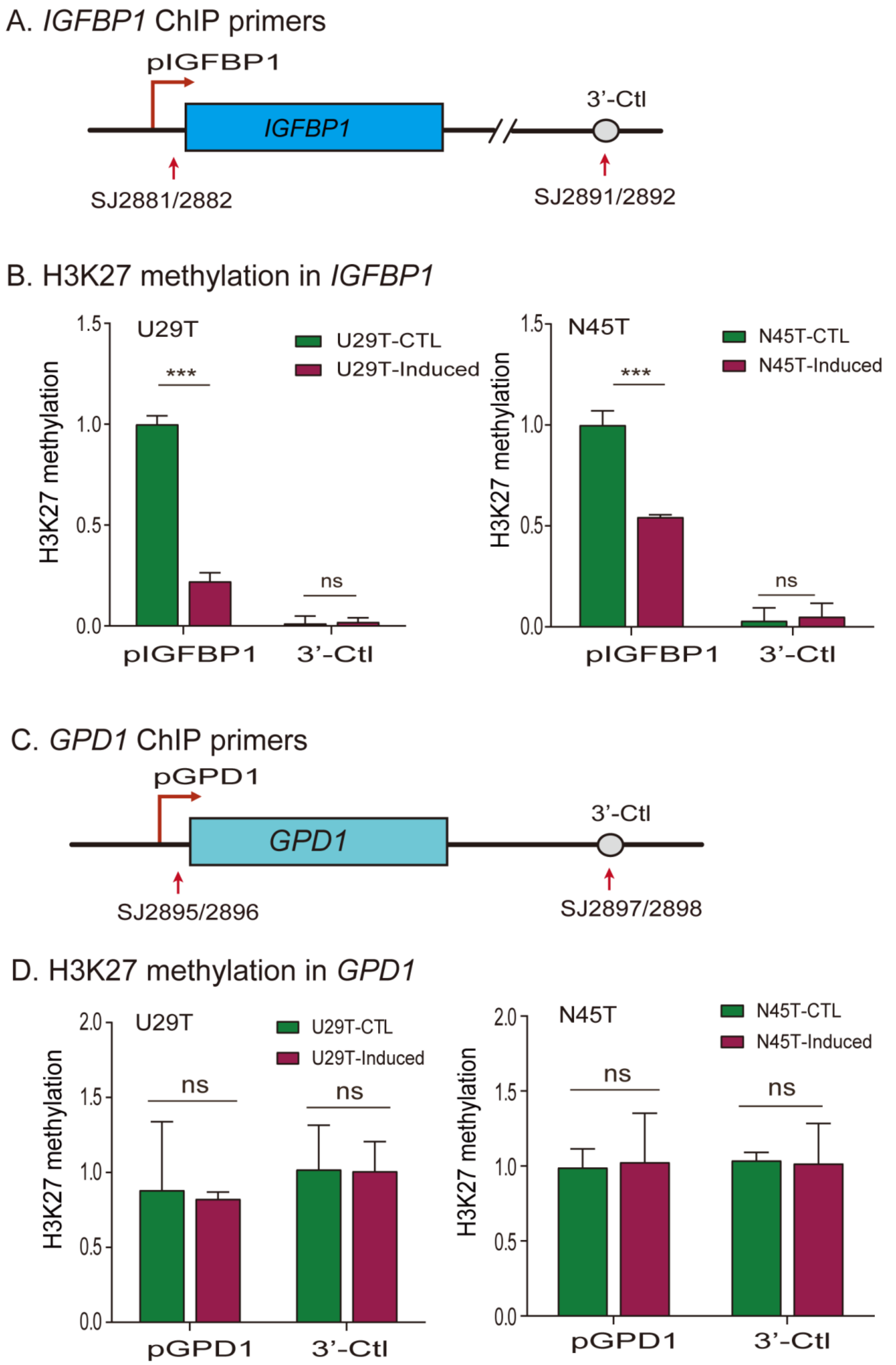

Figure S6. H3K27 methylation positive (*IGFBP1*) and negative (*GPD1*) controls. (**A**) Location of ChIP primers for the positive control *IGFBP1* gene. (**B**) Quantitation of the H3K27me3 ChIP signal of *IGFBP1*. Rabbit IgG was used as the control for data normalization. Error bars represent the standard error of the mean of three independent experiments. *** $p < 0.001$ as compared with control cells (CTL); ns: Not statistically significant. (**C**) Location of ChIP primers for the negative control *GPD1* gene. (**D**) Quantitation of the H3K27me3 ChIP signal of *GPD1*. Rabbit IgG was used as the control for data normalization. Error bars represent the standard error of the mean of three independent experiments. ns: Not statistically significant.

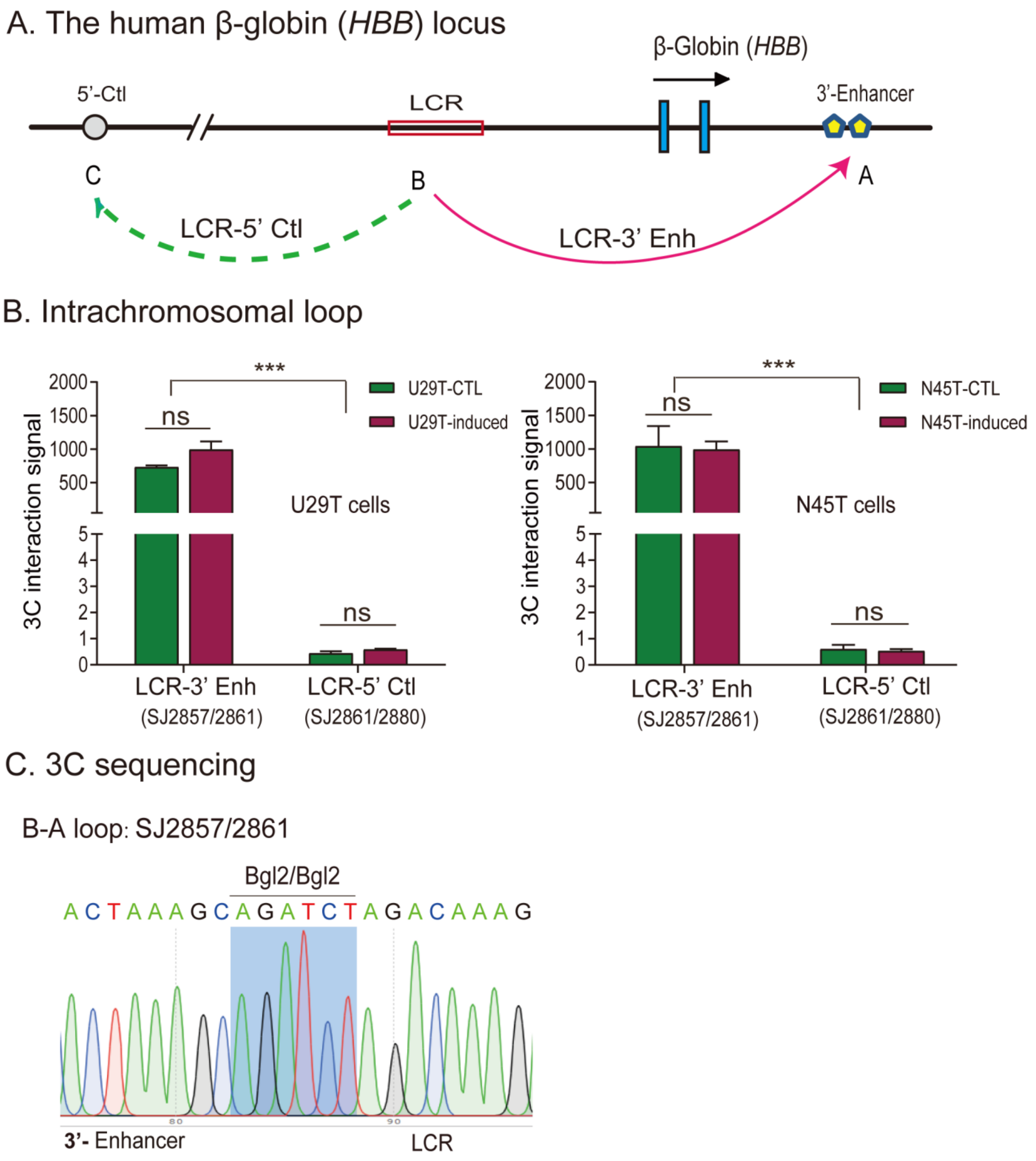

Figure S7. 3C positive control in the human β-Globin (*HBB*) locus. (**A**) Location of 3C primers for the positive control *HBB* gene. (**B**) Quantitation of intrachromosomal interaction in the *HBB* locus. Left panel: U29T cells; Right panel: N45T cells; 3′Enh: 3′-enhancer; 5′Ctl: 5′-negative control site; Induced: *in vitro* induction of decidualization; CTL: un-induced controls. Error bars represent the standard error of the mean of three independent experiments. *** $p < 0.001$ as compared with control cells (CTL); ns: Not statistically significant. (**C**) Sequencing of the *HBB* intrachromosomal loop 3C products. Blue background on the sequence: the 3C Bgl2 ligation site flanked by the LCR and the 3′-Enhancer.

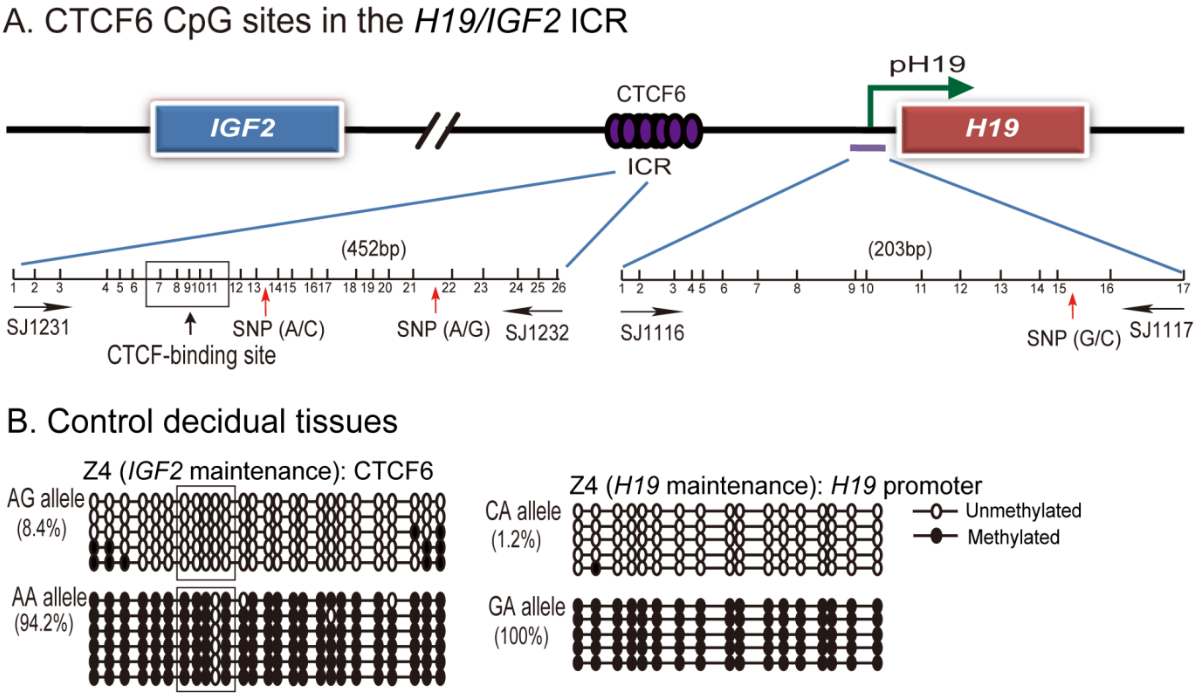

Figure S8. Allelic DNA methylation in the ICR of Z4 decidual tissues. (**A**) Schematic diagram of CpG islands in the *IGF2*/*H19* ICR. Locations of PCR primers are indicated by numbered arrows. Vertical lines: location of with CpG islands; Red arrows: single nucleotide polymorphisms allowing for discrimination of the two parental alleles. CTCF site 6 carrying CpG 7-11 is boxed. Genomic DNAs were extracted from decidual tissues. After treatment with sodium bisulfite, the *IGF2*/*H19* ICR DNA was amplified and sequenced. Open circles: unmethylated CpGs; Solid circles: methylated CpGs. (**B**) The methylation status at the CTCF6 site and the *H19* promoter in Z4 control decidual tissues. Numbers in parenthesis: percentage of methylated CpGs.

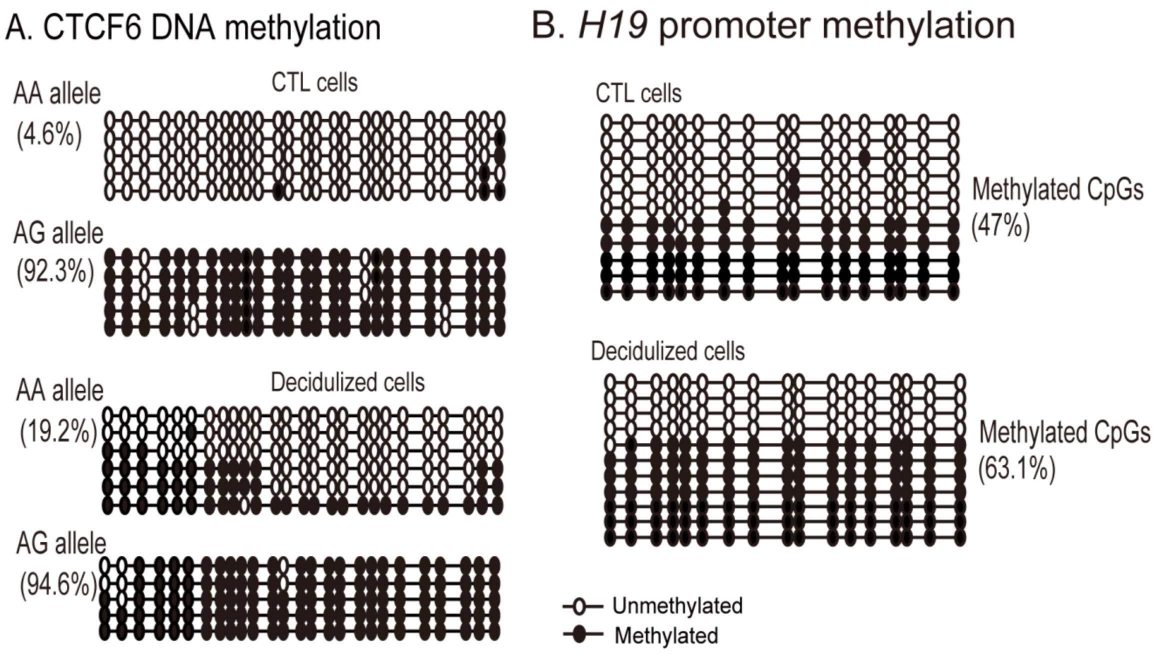

Figure S9. Allelic DNA methylation in the ICR of decidualized U29T cells. (**A**) Alteration of methylation status at the CTCF6 site in *in vitro* decidualized cells. After *in vitro* decidualization, cells were collected for DNA methylation analysis using sodium bisulfite sequencing. The two parental alleles were separated as the AA and AG alleles using the SNP at the CTCF6 site in the ICR. Numbers in the parenthesis are the percentage of methylated CpGs. Note the increment in methylated CpGs in the decidualized cells (19.2%) as compared with the control cells (4.6%). (**B**) DNA methylation of at the *H19* promoter following *in vitro* decidualization

Supplemental tables

Table S1. Oligonucleotide primers used for PCR.

ID	Oligo Name	Oligo sequence	Product size
RT-PCR			
H19	SJ182	GATCGGTGCCTCAGCG TTCG	115bp
	SJ183	GTCCTGCTTGTCACGTCCAC	
H19	SJ2854	ATCTGGAGTCTGGCAGGAGTG	246bp
	SJ184	TCAAACCCTGCCCACCAGCTC	
IGF2	J923	CTTGGACTTTGAGTCAAATTGGCCT	173bp
	J924	GAGGAGCCAGTCTGGGTTGTTGCTA	
IGF2	SJ2851	TGCGGCGGGGAGCTGGTGGAC	120bp
	SJ2852	GAAACAGCACTCCTCAACGATG	
IGFBP1	SJ1221	TATGATGGCTCGAAGGCTCT	158bp
	SJ1222	CCATTCTTGTTGCAGTTTGG	
PRL	SJ1223	AAACCAAACGGCTTCTAGAG	146bp
	SJ1224	GTTATAATAAGCAGAAAGGCGAGA	
EEF1A1	SJ3025	CAGGGACATCTCAGGCTGACT	126bp
	SJ3026	TCACACCCAGTGTGTAAGCCA	
β-actin	J880	CAGGTCATCACCATTGGCAATGAGC	135bp
	J881	CGGATGTCCACGTCACACTTCATGA	
DNA methylation			
H19 Promoter	SJ1116	GGGTTTGGGAGAGTTTGTGAGGT	225bp
	SJ1117	CAAACCGATTCCCATCCAATTAACC	
IGF2/H19 CTCF6	SJ1231	TGTTGAAGGTTGGGGAGATGGGA	452bp
	SJ1232	CCCAAACCATAACACTAAAACCCTC	
Imprinting expression			
H19	SJ179	CTTTACAACCACTGCACTACCTG	149bp
	SJ180	GCCATGAAGATGGAGTCGCCG	
H19	SJ179	CTTTACAACCACTGCACTACCTG	653bp
	SJ185	GCTTGAAGGCTGCTCCGTGATG	
IGF2	J923	CTTGGACTTTGAGTCAAATTGGCCT	173bp
	J924	GAGGAGCCAGTCTGGGTTGTTGCTA	
IGF2/H19 3C			
Promoter, 1st	SJ037	CACTGAGTC ATCTCAAAGT TAAGC	
Promoter, 2nd	SJ038	CCTTCAAG CACACTGCACACTCC	
Exon4, 1st	SJ1271	GCATCTGCTGCTGTCCCGCCG	
Exon4, 2nd	SJ1272	TGGCGCTCGTCTCCGGCTGC	
Exon4, 1st	SJ1273	CGCGGGCTAGAGGCACTTTAC	
Exon4, 2nd	SJ1274	GCGGGAGCGCCTCTCCTCCG	
CTCF6, 1st	SJ1118	TGTTGAAGGTTGGGGAGATGGG	

CTCF6, 2nd	SJ1275	GATGGCACGGAATTGGTTGTAG	
CTCF6, 1st	SJ1276	CATCACATAAGTAGGCGTGACTTG	
CTCF6, 2nd	SJ1277	CCCAGGCCATGACACTGAAGC	
CTCF6, 1st	SJ1278	TCCCAGAGCACAGCTCCGACTC	
CTCF6, 2nd	SJ1279	CCAGTGAACACTCTGATCTCCTC	
3’enhancer, 1st	SJ1280	CTGTCACCGCCAGCATGCTGTGG	
3’enhancer, 2nd	SJ1281	GAGGCCACCCCTCTTATGCCATG	
3’control	SJ1356	CACGGGCTTAGGAATGTGGTC	
5’control	SJ1357	CCATCCGCCAGAGACACTGC	
5’control	SJ1358	TCCCACTCAAGGCACCCCATG	
β-globin positive 3C			
Insulator, 1st	SJ2856	CAGTCAGTTCTTTGGACAAGTCT	
Insulator, 2nd	SJ2857	CTCTTCAGCCATCCCAAGACT	
enhancer, 1st	SJ2860	AGCCTCTGCATTGCCTTTACCG	
enhancer, 2nd	SJ2861	ATCCTCACGGTGACTAACGCA	
5’ control	SJ2880	GAAGCTCTCAATATGGCCTATG	
IGF2 ChIP			
5’ control	SJ1065	GCTGGGACTATAGGTGCGTG	173bp
	SJ1066	TGGCCCAGTGTGGTAGCTCA	
ChIP 1	JH3778	TCCATACAAGGAGGTGGGAA	121bp
	JH3779	AACCGGGAGCCCTGGACCATCCCGT	
ChIP 2	JH3780	TCTGTCTCCTACGAAGTCCCCAGAG	114bp
	JH3781	GAAGCCCTCCCTGTCCACGTCCTGA	
ChIP 3	JH3783	TGCCTGCCCGGAGACCCCAGCTCAC	115bp
	JH3784	CGCAGAGCGCCAAGGCCATGCTGAA	
IGFBP1 ChIP			
ChIP	SJ2881	CGTCATCCCCCTCCCAGCTGAG	231bp
	SJ2882	GCACAGGCCGCGCCACTTGCACC	
3’ control	SJ2891	GATACCACAACCAGACAAAGACAT	173bp
	SJ2892	ACCCCTGGAATAAATCCCACTG	
GPD1 ChIP			
ChIP	SJ2895	CACACCTGTCTGTACCCCTG	123bp
	SJ2896	CTCCACCACACGTGGGTGCA	
3’ control	SJ2897	CGCGCGATCCACCTCCAGA	139bp
	SJ2898	GTCTTTGGCGAGCCAAGCT	

Table S2. KEGG pathways that are associated with decidualization.

Pathway_ID	Term Des	Count	Fold Enrichment	*P* _ Value	-Log2 *P*
hsa04060	Cytokine-cytokine receptor interaction	34	4.027170825	7.69E-12	36.91978999
hsa04512	ECM-receptor interaction	11	3.639157409	7.84E-04	10.31773502
hsa04640	Hematopoietic cell lineage	11	3.639157409	7.84E-04	10.31773502
hsa04062	Chemokine signaling pathway	16	2.47590768	0.001896144	9.042716126
hsa04145	Phagosome	14	2.686359833	0.001992773	8.971006573
hsa05217	Basal cell carcinoma	8	4.264063226	0.002401621	8.701775832
hsa05332	Graft-versus-host disease	6	5.233168505	0.005105731	7.613666792
hsa04672	Intestinal immune network for IgA production	7	4.286744414	0.005267303	7.568719807
hsa04151	PI3K-Akt signaling pathway	22	1.835401128	0.008052501	6.956347429
hsa04550	Signaling pathways regulating pluripotency of stem cells	12	2.467065152	0.008994991	6.796662477

Table S3. Top 11 associated lncRNAs of decidual tissues by RNA sequencing from 3 RSA patients (Log2FoldChange > 7).

LncRNA	ENSEMBL	Locus	RSA Log2FoldChange	Regulation
MALAT1	ENSG00000251562	chr11:65497688-65506516	16.80970646	Up
H19	ENSG00000130600	chr11:1995176-2001470	15.10027518	Up
NEAT1	ENSG00000245532	chr11:65422774-65445540	14.39207199	Up
MIR4435-2HG	ENSG00000172965	chr2:111006015-111523376	10.28510655	Up
LINC00707	ENSG00000238266	chr10:6779549-6879450	9.905770556	Up
WT1-AS	ENSG00000183242	chr11:32435518-32458769	9.174663691	Up
LINP1	ENSG00000223784	chr10:6709530-6740532	7.932404414	Up
CYTOR	ENSG00000222041	chr2:87454781-87636740	7.910747049	Up
LINC00467	ENSG00000153363	chr1:211382736-211435570	7.409988851	Up
PLAC4	ENSG00000280109	chr21:41175231-41186788	7.255246685	Up
LUCAT1	ENSG00000248323	chr5:91054834-91314547	7.182273253	Up

Table S4. Basal characteristics of controls and RSA patients.

	**Controls**	**Cases**	***P* - value**
Age (years)	31.05 (8.13)	34.33 (7.4)	NS
BMI (cm/kg2)	22.8 (2.1)	21.3 (2.8)	NS
Clinical pregnancies (number)	2.25 ± 0.5	4.3 (2.2)	0.001
Live births (number)	1.25 ± 0.5	0	0.001
Artificial abortion (number)	n=57	0	0.001
Pregnancy losses (number)	0	n=32	0.001
Decidual tissues	n=57	n=32	
Tissue size (cm^3)	1 ± 0.5	1 ± 0.5	
Primary endometrial stromal cells	1	1	

All data are shown as mean ± SD. Comparison between the two groups (Control**s**/Cases) were performed by Student’s t-test.

Table S5. Genotype and allelic expression of *IGF2* and *H19*.

	Subjects	ID	*IGF2*			*H19*		
			Genotype	cDNA	Expression Mean ± SD	Genotype	cDNA	Expression Mean ± SD
Cases(N=32)	1	7	CC		0.1458±0.0181	AC	a	0.0153±0.0005
	2	8	CT	c/t	4.489±1.473	AC	a	0.1723±0.0381
	3	9	CT	t	17.4108±0.3992	AA		0.8891±0.0339
	4	22	CT	c/t	52.638±14.2263	AC	a/c	1.1458±0.1304
	5	25	CT	t	5.1738±0.0769	AA		0.1129±0.0006
	6	28	CC		0.1072±0.0093	AC	a	0.005±0.00045
	7	29	CC		0.1228±0.015	AC	c	0.011±0.0011
	8	E1	CT	c/t	0.8562±0.0665	AC	c	0.2104±0.0023
	9	E2	TT		0.5632±0.0277	AC	c	0.0314±0.0010
	10	E3	CT	c/t	30.8761±0.8955	AA		2.1312±0.0497
	11	E4	TT		1.1811±0.0542	AC	a	0.0944±0.0101
	12	E5	CT	c/t	0.7317±0.0841	AC	c	0.0393±0.0062
	13	E6	CC		0.8517±0.0343	AC	c	0.073±0.0058
	14	U1	CT	c	12.0754±0.4946	AC	a	0.2651±0.0141
	15	U2	CT	t	0.1322±0.012	AA		0.0201±0.0026
	16	U3	CT	t	0.127±0.0022	AA		0.0275±0.0026
	17	U4	CT	c	0.0153±0.0005	AC	c	0.0045±0.00029
	18	U5	CC		0.0111±0.0012	AC	c	0.0053±0.0001
	19	U11	CT	c/t	2.3202±0.2266	AA		0.2802±0.0304
	20	U13	TT		0.0634±0.0027	AC	a	0.0153±0.0009
	21	U14	CT	c/t	1.6466±0.1055	AA		0.2201±0.0089
	22	U15	CT	t	0.1404±0.0132	AC	a	0.0138±0.0018
	23	U17	CT	c/t	2.7935±0.4497	AA		0.2063±0.0076
	24	U18	CT	t	0.0626±0.0088	AC	a/c	0.0066±0.0008
	25	U19	CT	t	0.0814±0.0042	AA		0.0064±0.0004
	26	U20	CT	c/t	3.9371±1.3385	AC	a/c	0.5892±0.4222
	27	U21	TT		0.5046±0.0288	AC	a/c	0.0355±0.0013
	28	U22	CT	c	0.0262±0.0001	AA		0.0022±0.0003
	29	U23	CT	t	0.1058±0.0146	AC	a	0.0143±0.0018
	30	U25	CT	t	0.1153±0.0227	AC	a	0.0056±0.0007
	31	U28	CT	t	0.0714±0.0058	CC		0.0058±0.0009
	32	U29T*	CT	t	13.8314±0.2466	AC	c	0.6828±0.0730
Controls(N=57)	1	1	CT	c	0.2798±0.0098	AA		0.0241±0.0016
	2	2	CT	c/t	0.0291±0.0013	AA		0.0122±0.0002
	3	11	CT	t	0.0574±0.0008	CC		0.0041±0.0001
	4	13	CT	t	0.0928±0.0024	AC	a	0.0127±0.0009
	5	15	CT	t	0.0123±0.0013	AA		0.0051±0.0007
	6	16	CT	c/t	0.0156±0.0016	AA		0.005±0.0006
	7	20	CT	c/t	0.0563±0.0023	AA	a	0.009±0.0006
	8	Z1	CT	t	0.0959±0.0039	AA		0.0058±0.0004
	9	Z2	TT		0.0776±0.0009	CC		0.0193±0.0013
	10	Z3	CC		0.2201±0.0047	AA		0.0063±0.0008
	11	Z4	CT	t	0.8371±0.0824	AC	c	0.0154±0.0004
	12	Z5	TT		0.4809±0.0367	AC	a	0.0355±0.0038
	13	Z6	CT	t	1.6334±0.0593	AC	a/c	0.0536±0.0021
	14	N1	TT		0.2422±0.0113	AA		0.0169±0.0005
	15	N2	CT	c	0.031±0.0011	AA		0.0062±0.0003
	16	N3	TT		0.039±0.0018	AA		0.0027±0.0001
	17	N4	CT	t	0.184±0.0175	AC	a/c	0.0536±0.0049
	18	N5	TT		0.0279±0.002	AA		0.0085±0.0001
	19	N6	CT	t	0.0325±0.0009	AC	c	0.0177±0.0009
	20	N7	CT	t	0.0129±0	AC	a	0.0043±0.0003
	21	N8	CT	t	0.0513±0.0049	AC	c	0.014±0.0012
	22	N9	CT	c	0.023±0.0006	AA		0.0092±0.0057

23	N10	CT	t	1.4257±0.0678	AC	a/c	0.1336±0.0071
24	N11	CT	a	0.0164±0.0015	AA		0.0114±0.0004
25	N12	CT	t	0.021±0.0006	AC	a/c	0.0123±0.0012
26	N13	TT		0.1022±0.0077	AC	a	0.006±0.0001
27	N14	TT		0.1294±0.0135	AA		0.0368±0.0059
28	N15	CT	c	0.1858±0.0232	AA		0.0133±0.0065
29	N16	TT		0.2732±0.0034	AA		0.0192±0.0027
30	N17	CT	c	0.0593±0.0022	AA		0.0058±0.0016
31	N18	TT		0.0819±0.0056	AC	c	0.0034±0.0005
32	N19	TT		0.207±0.0152	AC	a/c	0.0843±0.0113
33	N20	CC		0.2655±0.0245	AC	c	0.0213±0.0052
34	N21	CT	c	0.9879±0.0462	CC		0.0816±0.0119
35	N22	CT	t	0.0333±0.0013	AC	c	0.0039±0.0002
36	N24	CT	t	0.1614±0.0401	AC	a	0.0515±0.0095
37	N25	TT		0.0205±0.0019	CC		0.0043±0.0003
38	N26	CC		0.0232±0.0017	AC	a	0.0163±0.0026
39	N27	CC		0.0476±0.0008	CC		0.0048±0.0001
40	N28	TT		0.0191±0.0032	AA		0.0058±0.0011
41	N29	TT		0.0242±0.0009	AA		0.0046±0.0006
42	N30	CT	t	1.7891±0.0862	AC	a	0.4206±0.0111
43	N31	CT	c	1.2382±0.0561	AA		0.3947±0.0208
44	N32	TT		0.1322±0.012	AA		0.0201±0.0026
45	N33	TT		0.0311±0.0017	AA		0.0093±0.0016
46	N34	CT	c	0.7324±0.0155	AA		0.2288±0.0092
47	N35	CT	c	0.4691±0.0254	AA		0.1401±0.0279
48	N36	TT		0.0374±0.0059	AC	a/c	0.0198±0.0029
49	N37	TT		0.0288±0.001	AA		0.0053±0.0009
50	N38	CT	t	0.0671±0.0071	AC	a	0.0159±0.0016
51	N39	CT	c	0.1805±0.0249	AA		0.0179±0.0020
52	N40	CT	c/t	0.0684±0.0028	AA		0.008±0.0003
53	N41	CC		0.0041±0.0005	AA		0.0048±0.0003
54	N42	CC		0.0958±0.0021	AA		0.0271±0.0208
55	N43	CT	t	0.0929±0.0101	AA		0.0218±0.0015
56	N44	CC		0.1111±0.0142	CC		0.0177±0.0018
57	N45T**	TT		0.0757±0.0094	AC	a	0.0064±0.0013

* Used for culturing U29T primary endometrial stromal cells. ** Used for culturing N45T primary endometrial stromal cells.
